# Effectiveness of a capacity-building training for knowledge and practices of tobacco control and oral cancer prevention: a pre-post study from rural northern India

**DOI:** 10.1186/s12903-026-08866-7

**Published:** 2026-06-29

**Authors:** Divya Khanna, Siva Santosh Kumar Pentapati, Priyanka Sharma, Tulika Shruti, Rajesh Vishawakarma, Anand Sharma, P Balasundaram, Niraj Kr Maurya, Vijay Kr Maurya, Rahul Kr Varma, Atul Budukh, Gauravi Mishra, Satyajit Pradhan, Pankaj Chaturvedi

**Affiliations:** 1 Dept. of Preventive Oncology, Mahamana Pandit Madan Mohan Malaviya Cancer Centre (MPMMCC) and Homi Bhabha Cancer Hospital (HBCH), Tata Memorial Centres, Varanasi, Uttar Pradesh 221005 India; 2 Homi Bhabha National Institute, Mumbai, India; 3 Departmentof Community Medicine, SVIMS - Sri Padmavathi Medical College for Women. Sri Venkateswara Institute of Medical Sciences (SVIMS), Tirupati, Andhra Pradesh 517501 India; 4 Department of Community Medicine, Preventive Oncology, ESIC Medical College and Hospital, Delhi, India; 5 Varanasi Cancer Registry, Mahamana Pandit Madan Mohan Malaviya Cancer Centre (MPMMCC) and Homi Bhabha Cancer Hospital (HBCH), Tata Memorial Centres, Varanasi, Uttar Pradesh 221005 India; 6 Centre for Cancer Epidemiology, Advanced Centre for Treatment, Research and Education in Cancer (ACTREC), Tata Memorial Centre, Mumbai, India; 7 Departmentof Preventive Oncology & Deputy Director, Centre for Cancer Epidemiology, Advanced Centre for Treatment, Research and Education in Cancer, Tata Memorial Centre, Mumbai, India; 8 Department of Radiation Oncology and Director, Mahamana Pandit Madan Mohan Malaviya Cancer Centre (MPMMCC) and Homi Bhabha Cancer Hospital (HBCH), Tata Memorial Centres, Varanasi, Uttar Pradesh 221005 India; 9 Department of Head Neck Surgery, Advanced Centre for Treatment, Research and Education in Cancer, Tata Memorial Hospital, Tata Memorial Centre, Mumbai, India

**Keywords:** Smokeless tobacco, Health education, COTPA Act, Areca nut, Counselling

## Abstract

**Background:**

Smokeless tobacco products and areca nut, widely used in Uttar Pradesh, are strongly associated with oral cancer, which ranks among the top three cancers in India. Data from the Population-Based Cancer Registry, Varanasi, highlights one of the highest oral cancer burdens across India. Despite the availability of national guidelines, frontline health workers (ASHAs) remain underutilised for tobacco cessation counselling and oral cancer symptom-based screening. This study assessed the impact of a structured training intervention on their knowledge, practices, and legal awareness.

**Methods:**

A pre–post intervention study was conducted in one rural block of Varanasi district between January and May 2023. A total of 202 ASHAs were enrolled through purposive sampling. The one-day training, based on Government of India modules, covered epidemiology, clinical features, determinants of tobacco use, cessation counselling, symptom-based oral cancer screening, and relevant legislation. Data were collected using a validated questionnaire before and after training. Descriptive statistics, Stuart–Maxwell tests, and Spearman’s correlations were applied for analysis.

**Results:**

Baseline scores revealed substantial knowledge and practice gaps: 45.6% of ASHAs had poor epidemiological awareness, 56.9% poor clinical knowledge, and 81.7% poor understanding of determinants of tobacco use. Only 19.8% reported routinely performing symptom-based screening for oral cancer, and while 64.8% “always” provided counselling, legal literacy was limited (only 38.1% aware of the COTPA Act). Following the intervention, significant improvements were observed across all domains (*p* < 0.01). Post-intervention most ASHAs reported willingness for oral cancer screening (64.8%) and provide tobacco cessation counselling (75.7%). Awareness of the COTPA Act increased to 65.4%. Strong positive correlations were observed between knowledge domains, suggesting synergistic gains.

**Conclusion:**

A structured, government-endorsed training programme significantly improved ASHAs’ knowledge, attitudes, and practices related to tobacco cessation and oral cancer screening, demonstrating the feasibility of leveraging frontline workers for community-based cancer prevention. However, achieving higher competency levels requires more intensive, competency-based training with supportive supervision. Integration within routine health systems and further research on long-term impact are warranted.

**Clinical trial registration number:**

Trial registration Clinical Trials Registry India CTRI/2021/02/031306. Date of registration 16/02/2021.

**Supplementary Information:**

The online version contains supplementary material available at https://doi.org/10.1186/s12903-026-08866-7.

## Introduction

Tobacco use is a major global health threat, responsible for more than eight million deaths annually, with the majority occurring in low- and middle-income countries [1].In India alone, tobacco use contributes to an estimated 1.35 million deaths each year, with smokeless tobacco (SLT) products such as gutkha, khaini, and betel quid being more widely consumed than smoked forms. These products often contain areca nut, a type I carcinogen, which further amplifies the risk of oral malignancies [2, 3].

Globally, oral cancer is among the most common cancers in South and Southeast Asia, reflecting the widespread use of SLT and areca nut in these regions [4]. In India, oral cancer ranks among the top three cancers in terms of incidence and mortality. Despite multiple regulations under the National Tobacco Control Programme (NTCP) and alignment with the WHO Framework Convention on Tobacco Control (FCTC), the burden remains unacceptably high [2, 5].

The challenge is particularly acute in Uttar Pradesh, one of India’s most populous states. Data from the Population-Based Cancer Registry (PBCR), Varanasi, reveal that oral cancer is the leading cancer site in the district in terms of both incidence and mortality [6]. Recent population-based cancer registry data from Varanasi (2017–2019) indicate that adolescents and young adults (15–39 years) constitute 16.2% (1105/6821) of all cancer cases, with an overall age-adjusted incidence rate of 21 per 100,000 population. Notably, oral cancers accounted for a substantial proportion (16.5%) of AYA cancers, with a truncated age adjusted incidence rate of 9.2 per 100,000 among males, ranking among the highest across Indian registries and much higher compared to several western countries [7]. The Global Youth Tobacco Survey (GYTS-India) further underscores this risk, reporting median ages of initiation as early as 9.9 years for SLT and 10.5 years for bidi smoking [8]. A cancer diagnosis at such a young age has profound implications, disrupting critical milestones of an individual’s life, such as education, career, family life, reproductive health, body image, and mental well-being. Beyond the individual, the burden extends to families, communities, and society at large, with economic and psychosocial consequences that persist for decades [7].

Frontline health workers, such as Accredited Social Health Activists (ASHAs), are strategically positioned as the first point of contact to address for delivering community-level tobacco-cessation counselling, generating awareness regarding the tobacco-related legislations and government funded programmes such as National Quitline. ASHAs also conduct symptom-based screening for oral cancer using the Community-Based Assessment Checklist (CBAC) as outlined in the population-based screening protocol under the National Programme for Prevention and Control of Cancer, Diabetes, Cardiovascular Diseases and Stroke (NPCDCS) framework [9]. The Government of India has also instituted performance-based incentives to support these activities, underscoring their programmatic importance [10].

CBAC includes symptoms such as the presence of a white or red patch in the oral cavity, intolerance to spicy food, restricted mouth opening, difficulty in tongue protrusion, change in voice (e.g., nasal tone), excessive salivation, and difficulty in chewing, swallowing, or speaking. Individuals reporting any of these symptoms are to be referred to Health and Wellness Centres for further oral cancer screening by a trained health worker, followed by appropriate diagnostic evaluation in accordance with established national guidelines [11].

Oral visual examination (OVE), when implemented systematically by trained workers, has been shown to reduce oral cancer morbidity and mortality in randomised community-based trials in India [12–15]. Likewise, tobacco cessation counselling delivered at the primary care or community level has been associated with increased quit attempts and reduced tobacco consumption. However, the coverage of oral cancer screening remains very low nationally (National Family Health Survey-5), and tobacco cessation counselling services are underutilised, particularly in high-burden states like Uttar Pradesh [16].

Despite the urgent need and the presence of scattered capacity-building interventions and national guidelines, there is a paucity of published evidence directly evaluating the effectiveness of such interventions for tobacco cessation counselling or oral cancer screening among frontline health workers in northern India, particularly in Varanasi [17–22]. Given the dual challenge of a high oral cancer burden and entrenched cultural acceptance of SLT and areca nut use, strengthening the knowledge and skills of frontline workers may represent a cost-effective and sustainable strategy to bridge gaps in prevention and early detection.

ASHA workers are a known face in the community and they play critical role in public health programmes. Hence, this study aimed to evaluate the impact of a structured capacity-building intervention on the knowledge, tobacco cessation counselling practices, and oral cancer screening practices of ASHA workers in Varanasi, using a pre–post study design.

## Materials and methods

### Study setting

Varanasi district, with a population of approximately four million, is administratively divided into eight rural blocks and 90 urban wards. According to the PBCR, the district bears a disproportionately high burden of oral cancer, with the age-standardised incidence and mortality rates of mouth cancer in men ranking second highest among the 38 PBCRs in India. This highlights the widespread tobacco use and underscores the urgent need for community-based tobacco control and cancer prevention initiatives in the region. The intervention was carried out in a randomly selected rural block of the district.

### Sample size

The sample size was estimated using the formula: N = [4*p(1-p)] / d2. Assuming 77% of the ASHAs would have poor knowledge [19] regarding the tobacco cessation and at a 95% confidence level and 9% relative error, the sample size was calculated as 148. Expecting a nonresponse rate of 10%, the final estimated sample size was 165. During the study period, we enrolled 202 ASHAs.

### Study design

The Department of Preventive Oncology at a Tertiary Cancer Centre (TCC) in Varanasi has been implementing tobacco-control activities as part of its ongoing community outreach programme for the prevention of oral, breast, and cervical cancers, in accordance with the NPCDCS and NTCP operational guidelines [9, 11, 23]. Within this framework, the present study was designed as a pre–post study focusing on capacity building of the frontline health workers (ASHAs), to strengthen tobacco-control efforts in Varanasi. The intervention was conducted between January and May 2023 in one randomly selected rural block of the district. All eligible ASHAs from this block were invited and enrolled for the training through purposive sampling during their administrative meetings at the Community Health Centres. The TCC obtained the necessary administrative approvals and has a memorandum of understanding (MoU) in place to carry out community-based cancer prevention activities across Varanasi.

Only de-identified data were analysed for this study. As per the Indian Council of Medical Research (ICMR) National Ethical Guidelines for Biomedical and Health Research Involving Human Participants (2017), programme evaluations conducted under public health programme of government agencies, where the purpose is refinement and monitoring of the programme and no individual identifiers are used, are considered exempt from formal ethics committee review.

### Training and study instrument

The training programme were one-day sessions with content focused on oral cancer screening, tobacco cessation counselling practices, epidemiology of oral cancer, clinical knowledge related to the effects of tobacco usage, facilitating factors and barriers to tobacco, and tobacco-related legislation. The content of the training programme has been derived from the National Health Mission Module for ASHA on Non-Communicable Diseases and National Tobacco Control Programme guidelines by the Government of India and was approved by two experts in the field [9, 11, 23]. Additionally, age and years of experience of ASHAs were recorded. Data was collected through a self-administered physical questionnaire, both for pre-test and post-test. A piloted questionnaire based on the module for ASHAs [9, 11, 23] was developed (supplementary information), validated by 2 experts in the field and was used for the pre-test and post-test survey (Supplementary information). A pilot study was conducted on 10 ASHAs before the main study to assess the questionnaire’s validity and reliability. The questionnaire’s reliability was assessed using Test-Retest. Kappa (k) = 0.81 and weighted Kappa (kW) = 0.83 were recorded. Internal consistency of questionnaires was assessed using Cronbach’s-Alpha, which yielded a result of 0.85.

### Intervention

The training intervention was delivered by the trained staff from the Preventive Oncology and Cancer Registry teams of a tertiary cancer centre. The training sessions were conducted in person at a designated Community Health Centres (CHCs) within the selected rural block of Varanasi district. ASHAs attended the sessions during their routine administrative meeting days to ensure feasibility and maximum participation. Each session was conducted as a one-day training programme, consisting of a structured 1.5-hour session that included a 30-minute pre-test, a 20-minute PowerPoint-based training, a 10-minute interactive discussion, and a 30-minute post-test. The PowerPoint presentations were developed from the ASHA training modules [9, 11, 23], adapted specifically for this study by two subject experts. The content was contextualised for ASHAs, incorporating both textual and infographic material to facilitate comprehension. The content was presented in the local language (Hindi) to ensure better comprehension. To ensure cultural and linguistic appropriateness, a pilot was conducted with 10 ASHAs to assess delivery in the local language and refine communication style. During the sessions, participants were actively encouraged to ask questions and clarify any concepts they found difficult.

### Statistical analysis

Data was entered in MS Excel. After cleaning, the data were imported into STATA 18.0, and the analysis was done. For analysis, the questionnaire items were grouped into three conceptual themes. Clinical Knowledge included questions on diseases caused by tobacco, the effects of passive smoking, and early symptoms of oral cancer. Epidemiological Awareness covered knowledge of cancer types linked to tobacco use and risk factors for oral cancer. Determinants of Tobacco Use captured participants’ knowledge of reasons for tobacco consumption and challenges in quitting.

Variables for these themes were constructed by adding the scores of the questions asked from the participants related to these themes. After getting the aggregated scores, under each theme, the scores were categorised arbitrarily as poor, average and good (Supplementary information).

Descriptive statistics were used to analyse the demographics of the participants, pre-test and post-test scores of the themes, tobacco legislation knowledge, attitude towards tobacco cessation counselling and oral cancer screening practices. Because we measured the same individuals before and after an intervention, and responses fall into multiple categories, we need a test that accounts for pairing and categorical outcomes. Hence, we used the Stuart–Maxwell test to compare the pre-test with the post-test data. As the scores are skewed, Spearman’s rank correlation was used to find the correlation between the pre-test and post-test scores of the three main themes. The association between participants’ sociodemographic characteristics and their pre- and post-test scores on epidemiological awareness, clinical knowledge, and determinants of tobacco use was assessed using Fisher’s exact test. The level of significance was set at 5%.

## Results

A total of 202 ASHAs were enrolled in the study. The mean (SD) age of the participants was 41.2 (5.9) years. Most participants were 40 years or younger (51.5%), hold secondary or senior secondary education qualifications (53%), and have more than 15 years of experience in the field work (50.5%). Detailed socio-demographic characteristics of the participants are given in Table 1.

**Table 1:** Socio-demographic characteristics of the participants (*N* = 202)

Characteristic	Frequency (*n*)	%
Age		
40 years or less	104	51.5
41–50 years	85	42.1
More than 50 years	13	6.4
Education		
Minimum Educational qualifications (completed class 8)	52	25.7
Secondary or Senior Secondary (class 9 to 12)	107	53
Graduate and above	43	21.3
Work experience		
5 years or less	31	15.4
6 to 10 years	32	15.8
11 to 15 years	37	18.3
More than 15 years	102	50.5

At baseline, most ASHAs recognised tobacco as a cause of oral cancer (87%), but far fewer identified alcohol (37%), sharp teeth or ill-fitting dentures (23%), and poor oral hygiene (8%) as risk factors. Cancer (62.4%) and lung diseases (54.4%) were the most identified tobacco-related diseases, while less than 10% were aware of associations with cataract, stroke, or infertility. Mouth cancer was most frequently reported as a tobacco-related cancer (87.6%), followed by cancers of the neck (38.1%) and lung (28.1%); very few identified bladder cancers (5.4%). Some ASHAs attributed tobacco to breast (28.2%), uterine (16.8%), and cervical cancers (26.7%) as well. Most ASHAs considered SLTs the most carcinogenic form (68.3%), but only one-fourth (27.2%) knew that areca nut is also a carcinogen. Regarding diseases associated with passive smoking, they most often cited lung disease/cancer (75.2%), heart disease (41.1%), and low birth weight (19.8%). Stress relief (54.9%) and peer pressure (47.0%) were viewed as key reasons for tobacco use, while addiction (78.2%) and lack of awareness (28.7%) were the main barriers to quitting. For signs and symptoms of oral cancer, ASHAs most frequently mentioned white/red patches (76.7%), difficulty opening the mouth (59.4%), and non-healing ulcers (53.5%) (Supplementary information).

At baseline, most ASHAs scored poorly across the three assessed domains: epidemiological awareness (45.6% classified as poor), clinical knowledge (56.9% poor) and determinants of tobacco use (81.7% poor). (Table 2) Although a majority reported a positive intent to practice screening as “always” providing tobacco-cessation counselling (64.8%), only one in five (19.8%) reported routinely performing symptom-based oral cancer screening. (Table 3) Regarding legal knowledge, while most ASHAs recognised that sale of tobacco to minors is prohibited (68.3%) and that vendors should not operate close to schools or other educational institutions (65.3%), substantial gaps existed for several statutory provisions: many were unaware of the COTPA Act itself (61.9%) and lacked knowledge about restrictions on public-place smoking (54.0%) and media advertising of tobacco (73.8%). (Table 4)

**Table 2:** Theme-wise comparison between the pre-test and post-test scores of participants (*N* = 202)

S.No	Theme/ categories (Score range)	Pre-test		Post-test		Chi-square (df)	*p*-value
		Freq (*n*)	%	Freq (*n*)	%		
1.	**Epidemiological Awareness**						
	Poor (Score = 0–4)	92	45.6	74	36.6	20.54 (2)	< 0.01
	Average (Score = 5–10)	95	47	97	48		
	Good (Score = 11–16)	15	7.4	31	15.4		
2.	**Clinical Knowledge**						
	Poor (Score = 0–7)	115	56.9	77	38.1	38.46 (2)	< 0.01
	Average (Score = 8–16)	78	38.6	91	45.1		
	Good (Score = 17–23)	9	4.5	34	16.8		
3.	**Determinants of Tobacco Use**						
	Poor (Score = 0–3)	165	81.7	130	64.4	24.65 (2)	< 0.01
	Average (Score = 4–6)	34	16.8	61	30.2		
	Good (Score = 7–9)	3	1.5	11	5.4		

**Table 3:** Comparison of intent to practice tobacco counselling and symptom-based screening between pre and post-intervention (*N* = 202)

Response	Tobacco counselling		Chi-square (df)	p-value	Oral cancer screening		Chi-square (df)	*p*-value
	Pre-test n (%)	Post-test n (%)			Pre-test n (%)	Post-test n (%)		
Never	30 (14.9)	22 (10.9)	11.63 (3)	0.01	82 (40.6)	27 (13.4)	85.45 (3)	< 0.01
Sometimes	15 (7.4)	4 (1.9)			62 (30.7)	26 (12.9)		
Mostly	26 (12.9)	23 (11.4)			18 (8.9)	18 (8.9)		
Always	131 (64.8)	153 (75.7)			40 (19.8)	131 (64.8)		

**Table 4:** Comparison of Tobacco legislation-related knowledge from pre to post (*N* = 202)

S.No	Question & categories	Pre-test		Post-test		Chi-square (df)	*p*-value
		Freq (*n*)	%	Freq (*n*)	%		
1.	**COTPA Act**						
	Knowledge present	77	38.1	132	65.4	40.48 (2)	< 0.01
	Knowledge absent	65	32.2	50	24.7		
	No response/ Don’t know	60	29.7	20	9.9		
2.	**Tobacco sale to minors (aged < 18 years)**						
	Correct Response	138	68.3	164	81.2	14.09 (2)	< 0.01
	Wrong Response	36	17.8	21	10.4		
	No Response/ Don’t know	28	13.9	17	8.4		
3.	**Proximity of sales of tobacco to schools/colleges**						
	Correct Response	132	65.3	144	71.3	10.89 (2)	< 0.01
	Wrong Response	30	14.9	39	19.3		
	No Response/ Don’t know	40	19.8	19	9.4		
4.	**Media advertisement restriction (tobacco)**						
	Correct Response	53	26.2	71	35.2	6.72 (2)	0.03
	Wrong Response	96	47.5	93	46		
	No Response/ Don’t know	53	26.2	38	18.8		

A significant improvement was observed in the knowledge and perceptions of frontline health workers for tobacco use following the capacity-building intervention (*p* < 0.01 across all domains). For epidemiological awareness, the proportion of participants with good awareness increased from 7.4% in the pre-test to 15.4% in the post-test, while those with poor awareness decreased from 45.6% to 36.6%. For clinical knowledge, the percentage of participants with good knowledge improved from 4.5% to 16.8%, accompanied by a reduction in poor knowledge from 56.9% to 38.1%. Similarly, for determinants of tobacco use, the proportion of participants with good scores increased from 1.5% to 5.4%, and with average scores from 16.8% to 30.2%, whereas those with poor scores declined from 81.7% to 64.4%. (Table 2)

Significant improvements were noted in attitude towards both tobacco cessation counselling and oral cancer symptom-based screening following the intervention. For tobacco cessation counselling, the proportion of frontline health workers who reported “always” providing counselling increased from 64.8% at baseline to 75.7% post-intervention. Correspondingly, those who reported “never” counselling declined from 14.9% to 10.9%, and “sometimes” counselling decreased from 7.4% to 1.9%. For screening, a substantial improvement was observed, with the proportion of participants who reported “always” screening rising from 19.8% to 64.8%. At the same time, those who reported “never” screening decreased sharply from 40.6% to 13.4%. (Table 3)

The intervention led to notable improvements in knowledge regarding tobacco legislation among frontline health workers. Awareness of the COTPA Act significantly increased from 38.1% at baseline to 65.4% post-intervention, while the proportion of those reporting no knowledge declined from 29.7% to 9.9%. Knowledge of the ban on tobacco sale to minors (< 18 years) also improved significantly, with correct responses increasing from 68.3% to 81.2%. Additionally, knowledge on the prohibition of tobacco sales near schools/colleges showed significant improvement (65.3% to 71.3%), and the knowledge of tobacco advertisement restrictions improved from 26.2% to 35.2%. (Table 4)

**Fig. 1:**
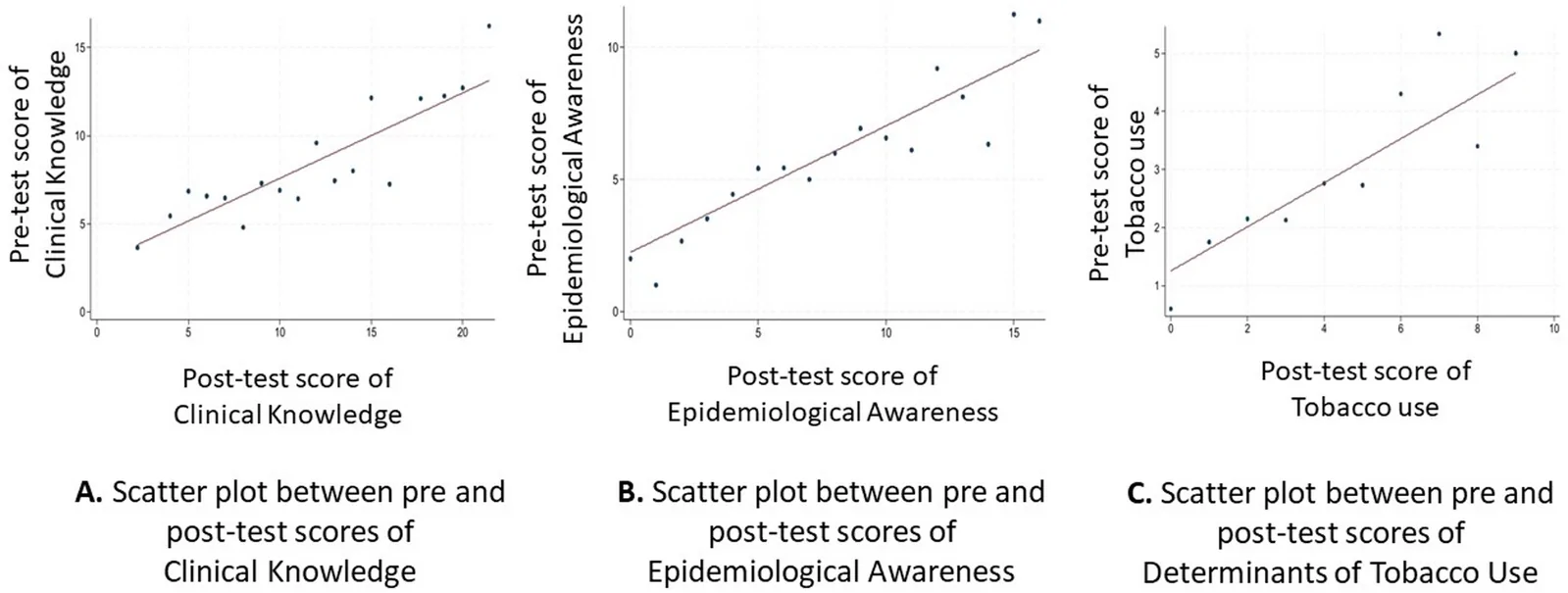
Scatter plots of Pre-test and Post-test scores of the themes

A significant positive correlation was observed between the pre- and post-test scores across all three domains-epidemiological awareness, determinants of tobacco use, and clinical knowledge (*p* < 0.01). The strongest correlation was noted between clinical knowledge and epidemiological awareness, with a correlation coefficient of 0.77 in the post-test and 0.72 in the pre-test. Moderate-to-strong correlations were also observed between clinical knowledge and determinants of tobacco use (Rho = 0.64 post-test; Rho = 0.57 pre-test). Similarly, epidemiological awareness and determinants of tobacco use showed moderate-to-strong correlations (Rho = 0.71 post-test; Rho = 0.54 pre-test). These findings indicate that participants with higher knowledge in one domain tended to score higher in the other, and improvements in one domain were associated with corresponding improvements in other domains following the intervention. (Table 5; Fig. 1)

**Table 5:** Correlation coefficient between the pre- and post-scores of all three themes

		Epidemiological awareness		Determinants of Tobacco Use		Clinical Knowledge	
		pre-test	post-test	pre-test	post-test	pre-test	post-test
Epidemiological awareness	pre-test	1					
	post-test	0.58*	1				
Determinants of Tobacco Use	pre-test	0.54*	0.45*	1			
	post-test	0.47*	0.71*	0.49*	1		
Clinical Knowledge	pre-test	0.72*	0.52*	0.57*	0.42*	1	
	post-test	0.56*	0.77*	0.40*	0.64*	0.58*	1

*p-value < 0.01

Post-intervention, educational status showed a significant association with epidemiological awareness, clinical knowledge and determinants of tobacco use scores, with greater improvements observed among participants with higher educational attainment. No association was observed with age or years of experience. (Supplementary information)

## Discussion

Varanasi’s high burden of oral cancer underscores the urgent need for community-centred prevention and early-detection strategies [6]. The early initiation of smokeless tobacco use and the socio-cultural acceptance of areca nut in this region continue to fuel risk, making frontline health workers critical agents for delivering preventive interventions [18, 24]. Frontline health workers (ASHAs) are uniquely positioned to deliver this response because they reach households routinely, are trusted by the community, and can integrate brief cessation counselling and opportunistic oral visual examination (OVE) into existing workflows [9]. Evidence from India and other LMICs supports task-shifting for both counselling and screening when frontline workers are adequately trained [25].

This study demonstrated substantial knowledge and practice gaps among ASHAs at baseline, particularly in their understanding of oral cancer risk factors, clinical signs, and tobacco-related legislation. While most participants recognized tobacco as a cause of oral cancer, far fewer identified other established risk factors such as alcohol consumption, areca nut use, poor oral hygiene, and ill-fitting dentures. This imbalance likely reflects the heavy emphasis of national communication campaigns on tobacco harms, with comparatively limited public health messaging on alcohol and oral hygiene as independent and synergistic risk factors for oral carcinogenesis. In settings like Varanasi, where areca nut is culturally embedded and oral hygiene practices are often suboptimal, such knowledge deficits may hinder effective community education [26, 27]. These findings highlight the need to broaden training content and community-focused educational efforts to include the full spectrum of oral cancer risk factors.

Awareness of the harms of passive smoking was also limited primarily to respiratory diseases, with little recognition of cardiovascular or reproductive consequences, a concerning gap given the ASHAs’ central role in maternal and child health.

The study baseline data revealed important gaps despite a generally positive response toward counselling. While 64.8% of ASHAs reported “always” providing tobacco-cessation counselling, large proportions scored poorly on core knowledge domains-69.3% on epidemiological awareness, 56.9% on clinical knowledge, and 81.7% on determinants of tobacco use, and only 19.8% (one in five) reported routinely performing symptom-based screening. This poor screening implementation is aligned with the national surveys as well as the local studies [27–29].

Legal literacy was similarly low, with many participants unaware of the COTPA Act and its provisions, particularly those related to media advertisement. This is concerning in the Indian context, where tobacco control efforts, despite being supported by one of the world’s largest publicly funded programmes, continue to face challenges in enforcement [30]. Evidence suggests that regulatory gaps are often exploited through practices such as surrogate advertising, twin packaging, and promotion of areca nut products [31].

In this setting, frontline health workers play a critical role in bridging policy and practice. Evidence indicates that involvement of healthcare providers can improve tobacco cessation outcomes; however, inadequate knowledge, limited promotion of cessation services, and social stigma may hinder their utilization, particularly in rural settings. Frontline workers such as ASHAs are central to health education, behaviour change communication, and referral pathways. Knowledge of COTPA provisions enables them to promote tobacco control measures at the community level, while awareness of Quitline services facilitates timely referral and improved utilization of government-supported cessation services [32].

The intervention substantially improved knowledge across epidemiological, clinical and behavioural-determinant domains, as well as understanding of tobacco legislation. By examining correlations across these domains, the study provides insights into how improvements in one area of knowledge may complement and reinforce others. Importantly, the training also appeared to positively influence attitudes toward tobacco-cessation counselling and screening, an essential component of sustained practice changes at the community level.

As India accelerates the scale-up of oral cancer screening programmes, digital innovations such as smartphone-based imaging, tele-medicine and AI-assisted image analysis offer opportunities to enhance diagnostic accuracy, reduce inter-observer variability, and support real-time decision-making in primary care [27, 33]. However, the effectiveness of such technologies depends on the readiness of frontline workers. Without adequate foundational knowledge and counselling skills, digital workflows may lead to low uptake, poor image quality, incorrect triage or referral, and potential loss of community trust. Therefore, before the wide scale introduction of AI and digital workflows, programmes must ensure baseline competency in tobacco control and oral-cancer recognition, and integrate digital-skill modules, supervised field practice and ongoing quality assurance into capacity-building packages so that technological innovations are both usable and impactful in the field [27, 34].

Although national guidelines and the CBAC outline training requirements and screening procedures, operational clarity on referral pathways remains limited. For screening programmes to translate into timely diagnosis and treatment, ASHAs require structured guidance on identifying appropriate referral facilities, ensuring follow-up, and facilitating navigation through the health system. Furthermore, their awareness of government-funded schemes, particularly the financial protection and treatment benefits available under Ayushman Bharat is likely to influence both community uptake of services and diagnostic and treatment compliance. Strengthening ASHAs’ understanding of such schemes should be an integral part of capacity-building packages.

This study contributes to the literature by applying government-endorsed training modules within a pre–post design to assess changes across multiple domains related to oral cancer prevention and tobacco cessation. The comprehensive assessment, including epidemiological, clinical, behavioural, practice-related and legal domains provides a multidimensional understanding of how training influences frontline worker readiness.

Several limitations merit consideration. Intent for counselling and screening practices were self-reported and may be influenced by social desirability bias. The post-test was administered immediately after the intervention, limiting the ability to assess long-term knowledge retention and sustained behavioural change. This structured, government-endorsed training programme substantially improved ASHAs’ knowledge, counselling, and screening practices. Yet, one-third still had poor knowledge of tobacco and oral cancer and very few (5.4%) had good knowledge of behavioural determinants. We did not collect information on tobacco use among ASHAs themselves, which may influence counselling behaviour. Furthermore, we did not inquire regarding the awareness of National Tobacco Quit line services. Additionally, community-level outcomes such as reductions in tobacco use or improvements in early detection could not be evaluated. Future research should include longitudinal follow-up and community-based trials to assess the durability of knowledge gains, translation into practice, and broader public health impact. In the Indian setting, frontline workers undertake a multitude of health service responsibilities; however, this study did not assess their perceived willingness to undertake cancer screening activities, which should be explored in future research.

Post-intervention, higher educational status was significantly associated with greater improvements in epidemiological awareness, clinical knowledge and determinants of tobacco use, suggesting differential uptake of training content based on educational background. No association was observed with age or years of experience. These findings indicate the need for tailored, competency-based training approaches, incorporating interactive methods such as case-based learning and skill-based sessions, to ensure more uniform effectiveness and sustained learning across frontline workers.

## Conclusion

This study demonstrates that a structured, government-endorsed training programme can significantly improve ASHAs’ knowledge, attitudes, and self-reported practices related to tobacco cessation and oral cancer screening. Despite notable baseline deficiencies in clinical knowledge, behavioural determinants, and legislative awareness, the intervention resulted in consistent improvements across all domains, supporting the feasibility of leveraging frontline workers for community-based cancer prevention. However, the relatively small proportion of participants attaining higher competency levels indicates the need for more intensive, iterative, and skill-oriented training approaches, supported by supervision and periodic assessment.

In high-burden settings such as Varanasi, strengthening frontline capacity remains critical to improving early detection and tobacco control efforts. Embedding such training within routine health system workflows, along with clear referral pathways and linkages to cessation services, may enhance programme effectiveness. Future research should focus on assessing long-term retention, sustained practice change, and broader programmatic impact.

## Supplementary Information

Supplementary Material 1.

## Data Availability

The datasets used and/or analysed during the current study are available from the corresponding author on reasonable request.

## Abbreviations

AI: Artificial Intelligence
ASHA: Accredited Social Health Activist
AYA: Adolescents and Young Adults
CBAC: Community-Based Assessment Checklist
CHC: Community Health Centre
COTPA: Cigarettes and Other Tobacco Products Act
CTRI: Clinical Trials Registry India
FCTC: Framework Convention on Tobacco Control
GoI: Government of India
GYTS: Global Youth Tobacco Survey
ICMR: Indian Council of Medical Research
k: Kappa Statistic
Kw: Weighted Kappa Statistic
LMICs: Low-and Middle-Income Countries
MoU: Memorandum of Understanding
NFHS: National Family Health Survey
NPCDCS: National Programme for Prevention and Control of Cancer, Diabetes, Cardiovascular Diseases and Stroke
NTCP: National Tobacco Control Programme
OVE: Oral Visual Examination

## References

[1] World Health Organization. Tobacco. 2025. https://www.who.int/news-room/fact-sheets/detail/tobacco. Accessed 21 Oct 2025.

[2] Swasticharan L, Arora M, Sinha P, Ray CS, Munish VG, Shrivastav R, editors. Report on Tobacco Control in India 2022. Vol 2. New Delhi: Ministry of Health and Family Welfare, Government of India. 2022. https://ntcp.mohfw.gov.in/assets/document/surveys-reports-publications/Report%20on%20Tobacco%20Control%20in%20India%202022_22%20April%202024.pdf. Accessed 21 Oct 2025.

[3] Bouvard V, Nethan ST, Singh D, Warnakulasuriya S, Mehrotra R, Chaturvedi AK, Chen TH, Ayo-Yusuf OA, Gupta PC, Kerr AR, Tilakaratne WM. IARC perspective on oral cancer prevention. N Engl J Med. 2022;387(21):1999–2005. 10.1056/nejmsr2210097. 36378601

[4] Bray F, Laversanne M, Sung H, Ferlay J, Siegel RL, Soerjomataram I, Jemal A. Global cancer statistics 2022: GLOBOCAN estimates of incidence and mortality worldwide for 36 cancers in 185 countries. CA Cancer J Clin. 2024;74(3):229–63. 10.3322/caac.21834. 38572751

[5] World Health Organization. WHO Framework Convention on Tobacco Control (WHO FCTC). https://www.who.int/europe/teams/tobacco/who-framework-convention-on-tobacco-control-(who-fctc) Accessed 21 Oct 2025.

[6] Budukh AM, Pradhan S, Singh VB, Khanna D, Bagal SS, Chakravarti PS, Sharma AN, Vishwakarma RK, Shinde SS, Khargekar NC, Chaturvedi P. Cancer pattern in Varanasi district from Uttar Pradesh state of India, a foundation for cancer control based on the first report of the population-based cancer registry. Indian J Cancer. 2024;61(2):383–9. 10.4103/ijc.ijc_44_21. 36861723

[7] Khanna D, Vishwakarma R, Sharma AN, Budukh A, Verma RK, Riguvanshi A, Mahmood F, Chaturvedi P, Pradhan S. Epidemiology of adolescent and young adult cancer and associated disparities in cancer pattern and care in India: findings from Varanasi’s population-based cancer registry, 2017–2019. Cancer Causes Control. 2025. 10.1007/s10552-025-02030-2. 40652115 PMC12578687

[8] International Institute for Population Sciences (IIPS), Ministry of Health and Family Welfare (MoHFW). Global Youth Tobacco Survey (GYTS-4), India 2019: Report. Mumbai: IIPS. 2021. https://ntcp.mohfw.gov.in/assets/document/surveys-reports-publications/GYTS%204%20Final%20Report.pdf. Accessed 21 Oct 2025.

[9] Ministry of Health and Family Welfare. Module for ASHA on non-communicable diseases. New Delhi: Government of India. https://mohfw.gov.in/sites/default/files/Module%20for%20ASHA%20on%20Non-communicable%20Diseases_1.pdf. Accessed 21 Oct 2025.

[10] Department of Health and Family Welfare. ASHA incentives. https://nhm.gov.in/New-Update-2023-24/ASHA/Orders_and_guidelines/ASHA-INCENTIVES.pdf. Accessed 21 Oct 2025.

[11] National Health Systems Resource Centre. Training manual on oral care for ASHA: at Ayushman Bharat – Health and Wellness Centres. New Delhi: NHSRC. 2021. https://nhsrcindia.org/sites/default/files/2021-12/Oral%20Care%20Training%20Manual%20for%20ASHA.pdf. Accessed 21 Oct 2025.

[12] Thampi V, Hariprasad R, John A, Nethan S, Dhanasekaran K, Kumar V, Birur P, Thakur JS, Lilford R, Rajpoot NM, Gill P. Feasibility of training community health workers in the detection of oral cancer. JAMA Netw Open. 2022;5(1):e2144022. 10.1001/jamanetworkopen.2021.44022. 35040966 PMC8767429

[13] Birur NP, Song B, Sunny SP, Mendonca KG, Mukhia P, Li N, Patrick S, AR SGS, Imchen S. Field validation of deep learning based point-of-care device for early detection of oral malignant and potentially malignant disorders. Sci Rep. 2022;12(1):14283. 10.1038/s41598-022-18249-x. 35995987 PMC9395355

[14] Sankaranarayanan R, Ramadas K, Thara S, Muwonge R, Thomas G, Anju G, Mathew B. Long term effect of visual screening on oral cancer incidence and mortality in a randomized trial in Kerala, India. Oral Oncol. 2013;49(4):314–21. 10.1016/j.oraloncology.2012.11.004. 23265945

[15] Gurushanth K, Sunny SP, Kuriakose MA, Birur PN. Feasibility, reliability, and effectiveness of oral cancer screening in South Asia and Southeast Asian countries: a systematic review and meta-analysis. Oral Dis. 2025;31(5):1490–502. 38817091 10.1111/odi.15001

[16] Government of Uttar Pradesh, Planning Department. Uttar Pradesh NFHS-5 fact sheet. Lucknow: Government of Uttar Pradesh. https://planning.up.nic.in/Go/SDG/Uttar_Pradesh_NFHS-5%20fact%20sheet.pdf. Accessed 21 Oct 2025.

[17] Ragavane P, Kengadaran S. Frontline health workers improves tobacco cessation through health literacy. J Fam Med Prim Care. 2023;12(7):1468–9. 10.4103/jfmpc.jfmpc_100_23. PMC10465054 37649764

[18] Gautam JK, Anand PK, Chouhan M, Babu BV. Capacity building of community health officers for optimizing the screening and early diagnosis of oral cancer in Jodhpur, Rajasthan, India. Sci Rep. 2025;15(1):16027. 10.1038/s41598-025-00225-w. 40341602 PMC12062443

[19] Garg A, Sinha A, Kumar N, Singh A, Akhtar S, Singh PK. Awareness about role of health literacy and self efficacy in tobacco cessation among primary health care workers: a quantitative questionnaire study. J Fam Med Prim Care. 2022;11(11):7036–41. 10.1108/IJSE-11-2016-0303. PMC10041204 36993129

[20] Panda R, Srivastava S, Persai D, Mathur MR, Modi B, Dave P, Arora M. Preparedness of frontline health workers for tobacco cessation: an exploratory study from two states of India. J Fam Med Prim Care. 2015;4(3):298–304. 10.4103/2249-4863.161301. PMC4535082 26288762

[21] Philip PM, Nayak P, Philip S, Parambil NA, Duraisamy K, Balasubramanian S. Population-based cancer screening through community participation: outcome of a district wide oral cancer screening programme from rural Kannur, Kerala, India. South Asian J Cancer. 2018;7(4):244–8. 10.4103/sajc.sajc_104_17. 30430093 PMC6190395

[22] Jhanjee S, Varshney MK, Panda UK, Kaloiya GS, Dayal P, Yadav D. Evaluation of a training workshop on tobacco cessation: capacity building initiative in India. Tob Induc Dis. 2018;16(1). 10.18332/TID/84169.

[23] Ministry of Health and Family Welfare. National Tobacco Control Programme: a guide for health care workers helping tobacco users quit. https://ntcp.mohfw.gov.in/assets/document/Guideline-manuals/A%20Guide%20for%20Health%20worker…Helping%20tobacco%20users%20quit.pdf. Accessed 21 Oct 2025.

[24] World Health Organization. Smokeless tobacco (SLT) products, WHO FCTC. 2018 Jan 10. https://extranet.who.int/fctcapps/fctcapps/fctc/kh/slt/news/smokeless-tobacco-slt-products. Accessed 21 Oct 2025.

[25] Joshi R, Alim M, Kengne AP, Jan S, Maulik PK, Peiris D, Patel AA. Task shifting for non-communicable disease management in low and middle income countries – a systematic review. PLoS ONE. 2014;9(8):e103754. 10.1371/journal.pone.0103754. 25121789 PMC4133198

[26] Khanna D, Shruti T, Tiwari M, Sharma P, Khan A, Ranjan S, Balasundaram P, Khargekar N, Chaturvedi P, Mishra A. Prevalence of oral potentially malignant lesions, tobacco use, and effect of cessation strategies among solid waste management workers in Northern India: a pre-post intervention study. BMC Oral Health. 2024;24(1):1292. 10.1186/s12903-024-05087-8. 39462398 PMC11515233

[27] Khanna D, Mishra A, Birur P, Shruti T, Gurushanth K, Mukhia N, Pathak R, Gurmeet Singh A, Shetty A, Pradhan S, Chaturvedi P. A prospective study on diagnostic accuracy of technology-enabled early detection of oral cancer and epidemiology of tobacco and other substances use in rural India. Cancer. 2025;131(1):e35702. 10.1002/cncr.35702. 39748663 PMC11695980

[28] International Institute for Population Sciences (IIPS), ICF. National Family Health Survey (NFHS-5), 2019–21. Mumbai: IIPS. 2021. https://dhsprogramme.com/pubs/pdf/FR375/FR375.pdf. Accessed 21 Oct 2025.

[29] Sharma P, Khanna D, Pradhan S, Birur P. Community cancer screening at primary care level in Northern India: determinants and policy implications for cancer prevention. Fam Med Community Health. 2023;11(Suppl 1):e002397. 10.1136/fmch-2023-002397. 38105243 PMC10729271

[30] Shruti T, Khanna D, Khan A, Dandpat A, Tiwari M, Singh AG, Mishra A, Shetty A, Birur P, Chaturvedi P. Status and determinants of early detection of oral premalignant and malignant lesions in India. Cancer Control. 2023;30:10732748231159556. 10.1177/10732748231159556. 36809192 PMC9947682

[31] Adhikari K, Pednekar MS, Stepanov I, Singh A, Nikam S, Singhavi H, Gota V, Ahluwalia JS, Chaturvedi P, Gupta PC, Khariwala SS. Observed circumvention of the gutkha smokeless tobacco ban in Mumbai, India. Tob Regul Sci. 2020;6(5):331. 10.18001/trs.6.5.3. 34676280 PMC8528234

[32] Bhatt G, Goel S, Grover S, Medhi B, Jaswal N, Gill SS, Singh G. Feasibility of tobacco cessation intervention at non-communicable diseases clinics: a qualitative study from a North Indian state. PLoS ONE. 2023;18(5):e0284920. 10.1371/journal.pone.0284920. 37141319 PMC10159160

[33] Ministry of Electronics and Information Technology. IndiaAI and National Cancer Grid launch CATCH grant programme to support AI innovations in cancer screening, diagnosis, and treatment. New Delhi: Press Information Bureau. 2025. https://www.pib.gov.in/PressReleasePage.aspx?PRID=2154341. Accessed 21 Oct 2025.

[34] do Nascimento IJ, Abdulazeem HM, Vasanthan LT, Martinez EZ, Zucoloto ML, Østengaard L, Azzopardi-Muscat N, Zapata T, Novillo-Ortiz D. The global effect of digital health technologies on health workers’ competencies and health workplace: an umbrella review of systematic reviews and lexical-based and sentence-based meta-analysis. Lancet Digit Health. 2023;5(8):e534–44. 10.1016/S2589-7500(23)00092-4. 37507197 PMC10397356

[35] Khanna D, Sharma P, Budukh A, Vishwakarma R, Sharma AN, Bagal S, Tripathi V, Maurya VK, Chaturvedi P, Pradhan S. Rural-urban disparity in cancer burden and care: findings from an Indian cancer registry. BMC Cancer. 2024;24(1):308. 10.1186/s12885-024-12041-y. 38448839 PMC10916062

[36] Indian Council of Medical Research. National ethical guidelines for biomedical and health research involving human participants. New Delhi: ICMR. 2017. https://ethics.ncdirindia.org/asset/pdf/ICMR_National_Ethical_Guidelines.pdf. Accessed 21 Oct 2025.

