# Supplementary material for: Effectiveness of a capacity-building training for knowledge and practices of tobacco control and oral cancer prevention: a pre-post study from rural northern India
Source: BMC Oral Health. 2026 Jun 29;26:1734. doi: 10.1186/s12903-026-08866-7 (PMC13576245; doi:10.1186/s12903-026-08866-7)
Supplement: Supplementary file 1 [file 12903_2026_8866_MOESM1_ESM.docx]

**Supplementary information checklist**

1. **Table-1 Distribution of correct responses to the questions in pre-test and post-test under the themes (Page 2)**
2. **Table-2 Scoring criteria for deciding the categories under each theme (Page 4)**
3. **Table-3 Association between Sociodemographic characteristics of the participants and Epidemiological Awareness Scores (Page 5)**
4. **Table-4 Association between Sociodemographic characteristics of the participants and Clinical Knowledge Scores (Page 6)**
5. **Table-5 Association between Sociodemographic characteristics of the participants and Determinants of Tobacco use Scores (Page 7)**
6. **Self-administered questionnaire (Page 8)**

**Supplementary information**

**Table-1 Distribution of correct responses to the questions in pre-test and post-test under the themes**

| **No.** | **Question** | **Correct Response – Pre-test** | | **Correct Response – Post-test** | | **p-value** |
| --- | --- | --- | --- | --- | --- | --- |
|  |  | **n** | **%** | **n** | **%** |  |
| **Theme 1: Epidemiological Awareness** | | | | | | |
| ***1*** | ***Risk factors for oral cancer*** | | | | | |
|  | Alcohol consumption | 75 | 37.13 | 77 | 38.12 | 0.79 |
|  | Use of tobacco | 175 | 86.63 | 180 | 89.11 | 0.38 |
|  | Family history of cancer | 33 | 16.34 | 27 | 13.37 | 0.33 |
|  | Poor oral hygiene | 17 | 8.42 | 36 | 17.82 | <0.01 |
|  | Sharp teeth and ill-fitting dentures | 46 | 22.77 | 47 | 23.27 | 0.88 |
|  | Unhealthy diet (low intake of fresh fruits and vegetables) | 6 | 2.97 | 15 | 7.43 | 0.04 |
| ***2*** | ***Types of cancer caused by tobacco use*** | | | | | |
|  | Mouth | 177 | 87.62 | 182 | 90.1 | 0.38 |
|  | Larynx (voice box) | 46 | 22.77 | 77 | 38.12 | <0.01 |
|  | Esophagus (food pipe) | 44 | 21.78 | 77 | 38.12 | <0.01 |
|  | Lungs | 57 | 28.22 | 84 | 41.58 | <0.01 |
|  | Stomach | 22 | 10.89 | 29 | 14.36 | 0.21 |
|  | Throat | 77 | 38.12 | 85 | 42.08 | 0.28 |
|  | Bladder | 11 | 5.45 | 23 | 11.39 | 0.02 |
| ***3*** | ***Types of tobacco that causes cancer*** | | | | | |
|  | Smoking tobacco (e.g., Cigarette/Bidi/Hookah) | 100 | 49.5 | 130 | 64.36 | <0.01 |
|  | Chewing tobacco (e.g., Gutkha/Khaini/Zarda/Pan Masala) | 138 | 68.32 | 161 | 79.7 | <0.01 |
|  | Areca nut (Supari) | 55 | 27.23 | 78 | 38.61 | <0.01 |
| **Theme 2: Clinical knowledge** | | | | | | |
| ***4*** | ***Tobacco use can cause the following diseases*** | | | | | |
|  | Asthma/Respiratory diseases | 110 | 54.46 | 128 | 63.37 | <0.01 |
|  | Cataract | 13 | 6.44 | 30 | 14.85 | <0.01 |
|  | Cancer | 126 | 62.38 | 146 | 72.28 | 0.01 |
|  | Increased risk of TB | 79 | 39.11 | 85 | 42.08 | 0.41 |
|  | Heart attack | 40 | 19.8 | 75 | 37.13 | <0.01 |
|  | Stroke | 19 | 9.41 | 54 | 26.73 | <0.01 |
|  | High blood pressure (BP) | 28 | 13.86 | 83 | 41.09 | <0.01 |
|  | Diabetes | 29 | 14.36 | 55 | 27.23 | <0.01 |
|  | Gum diseases | 31 | 15.35 | 65 | 32.18 | <0.01 |
|  | Low birth weight in newborns | 23 | 11.39 | 40 | 19.8 | 0.01 |
|  | Impotence and reduced fertility | 10 | 4.95 | 21 | 10.40 | 0.03 |
| ***5*** | ***Passive smoking (inhaling smoke from others' cigarettes) causes which of the following diseases?*** | | | | | |
|  | Heart disease | 83 | 41.09 | 113 | 55.94 | <0.01 |
|  | Lung disease/cancer | 152 | 75.25 | 164 | 81.19 | 0.09 |
|  | Premature birth in babies | 40 | 19.8 | 56 | 27.72 | 0.01 |
| ***6*** | ***Symptoms of oral cancer*** | | | | | |
|  | White/red patches in the mouth | 155 | 76.73 | 176 | 87.13 | <0.01 |
|  | Non-healing ulcers/wounds | 108 | 53.47 | 121 | 59.9 | 0.09 |
|  | White patches on the inner cheeks | 85 | 42.08 | 109 | 53.96 | <0.01 |
|  | Difficulty eating spicy food | 64 | 31.68 | 96 | 47.52 | <0.01 |
|  | Difficulty opening the mouth | 120 | 59.41 | 131 | 64.85 | 0.17 |
|  | Difficulty sticking out the tongue | 69 | 34.16 | 95 | 47.03 | <0.01 |
|  | Voice changes | 68 | 33.66 | 92 | 45.54 | <0.01 |
|  | Excessive salivation | 49 | 24.26 | 62 | 30.69 | 0.06 |
|  | Difficulty chewing/swallowing/speaking | 62 | 30.69 | 89 | 44.06 | <0.01 |
| **Theme 3: Determinants of tobacco use** | | | | | | |
| ***7*** | ***Why do people use tobacco?*** | | | | | |
|  | For pleasure | 28 | 13.86 | 52 | 25.74 | <0.01 |
|  | To relieve stress | 111 | 54.95 | 117 | 57.92 | 0.44 |
|  | Under peer pressure | 95 | 47.03 | 121 | 59.9 | <0.01 |
|  | To appear mature | 5 | 2.48 | 39 | 19.31 | <0.01 |
|  | Out of curiosity | 15 | 7.43 | 31 | 15.35 | <0.01 |
| ***8*** | ***Why do people find it difficult to quit tobacco?*** | | | | | |
|  | Addiction | 158 | 78.22 | 153 | 75.74 | 0.51 |
|  | Lack of awareness about harmful effects | 58 | 28.71 | 82 | 40.59 | <0.01 |
|  | No current health problems | 11 | 5.45 | 22 | 10.89 | 0.03 |
|  | No counseling by health professionals | 13 | 6.44 | 18 | 8.91 | 0.33 |

**Table-2 Scoring criteria for deciding the categories under each theme**

|  | **Theme/ Category** | **Score** |
| --- | --- | --- |
| **1** | ***Epidemiological Awareness*** | **0-16** |
|  | Poor | 0-4 |
|  | Average | 5-10 |
|  | Good | 11-16 |
| **2** | ***Clinical Knowledge*** | **0-23** |
|  | Poor | 0-7 |
|  | Average | 8-16 |
|  | Good | 17-23 |
| **3** | ***Determinants of Tobacco use*** | **0-9** |
|  | Poor | 0-3 |
|  | Average | 4-6 |
|  | Good | 7-9 |

**Table-3 Association between Sociodemographic characteristics of the participants and Epidemiological Awareness Scores**

|  | **Pre-test** | | | | | **Post-test** | | |
| --- | --- | --- | --- | --- | --- | --- | --- | --- |
|  | **Poor**  **(%)** | **Average**  **(%)** | **Good**  **(%)** | **p-value** | **Poor**  **(%)** | **Average**  **(%)** | **Good**  **(%)** | **p-value** |
| **Age (in years)** | | | |  |  | | |  |
| 40 years or less | 48 (46.15) | 50 (48.08) | 6 (5.77) | 0.30 | 38 (36.54) | 54 (51.92) | 12 (11.54) | 0.18 |
| 41-50 | 40 (47.06) | 39 (45.88) | 6 (7.06) |  | 32 (37.65) | 39 (45.88) | 14 (16.47) |  |
| More than 50 | 4 (30.77) | 6 (46.15) | 3(23.08) |  | 4 (30.77) | 4 (30.77) | 5 (38.46) |  |
| **Education** | | | |  |  | | |  |
| Minimum Educational qualifications (completed class 8) | 28 (53.85) | 20 (38.46) | 4 (7.69) | 0.37 | 22 (42.31) | 25 (48.08) | 5 (9.62) | **0.05** |
| Secondary or Senior Secondary (class 9 to 12) | 48 (44.86) | 53 (49.53) | 6 (5.61) |  | 43 (40.19) | 50 (46.73) | 14 (13.08) |  |
| Graduate and above | 16 (37.21) | 22 (51.16) | 5 (11.63) |  | 9 (20.93) | 22 (51.16) | 12 (27.91) |  |
| **Work experience (in years)** | | | |  |  | | |  |
| 5 or less | 3 (37.5) | 4 (50) | 1 (12.5) | 0.80 | 1 (12.5) | 6 (75) | 1 (12.5) | 0.22 |
| 6 – 10 | 13 (40.62) | 16 (50) | 3(9.38) |  | 9 (28.12) | 15 (46.88) | 8 (25) |  |
| 11 – 15 | 17 (45.95) | 17 (45.95) | 3 (8.11) |  | 18 (48.65) | 14(37.84) | 5 (13.51) |  |
| > 15 | 53 (51.96) | 43 (42.16) | 6 (5.88) |  | 40 (39.22) | 50 (49.02) | 12 (11.76) |  |

**Table-4 Association between Sociodemographic characteristics of the participants and Clinical Knowledge Scores**

|  | **Pre-test** | | | | | **Post-test** | | | |
| --- | --- | --- | --- | --- | --- | --- | --- | --- | --- |
|  | **Poor** | **Average** | **Good** | **p-value** | **Poor** | | **Average** | **Good** | **p-value** |
| **Age (in years)** | | | |  | | | |  |  |
| 40 years or less | 61 (58.65) | 39 (37.5) | 4 (3.85) | 0.33 | 39 (37.5) | | 49 (47.12) | 16 (15.38) | 0.13 |
| 41-50 | 49 (57.65) | 33 (38.82) | 3 (3.53) |  | 35 (41.18) | | 38 (44.71) | 12 (14.12) |  |
| More than 50 | 5 (38.46) | 6 (46.15) | 2 (15.38) |  | 3 (23.08) | | 4 (30.77) | 6 (46.15) |  |
| **Education** | | | |  | | | |  |  |
| Minimum Educational qualifications (completed class 8) | 37 (71.15) | 14 (26.92) | 1 (1.92) | 0.12 | 25 (48.08) | | 24 (46.15) | 3 (5.77) | **0.049** |
| Secondary or Senior Secondary (class 9 to 12) | 58 (54.21) | 44 (41.12) | 5 (4.67) |  | 38 (35.51) | | 50 (46.73) | 19 (17.76) |  |
| Graduate and above | 20 (46.51) | 20 (46.51) | 3 (6.98) |  | 14 (32.56) | | 17 (39.53) | 12 (27.91) |  |
| **Work experience (in years)** | | | |  | | | |  |  |
| 5 or less | 13 (41.94) | 17 (54.84) | 1 (3.23) | 0.14 | 9 (29.03) | | 16 (51.61) | 6 (19.35) | 0.74 |
| 6 – 10 | 18 (56.25) | 11 (34.38) | 3 (9.38) |  | 10 (31.25) | | 15 (46.88) | 7 (21.88) |  |
| 11 – 15 | 18 (48.65) | 17 (45.95) | 2 (5.41) |  | 17 (45.95) | | 14 (37.84) | 6 (16.22) |  |
| > 15 | 66 (64.71) | 33 (32.35) | 3 (2.94) |  | 41 (40.2) | | 46 (45.1) | 15 (14.71) |  |

**Table-5 Association between Sociodemographic characteristics of the participants and Determinants of Tobacco use Scores**

|  | **Pre-test** | | | | | **Post-test** | | | |
| --- | --- | --- | --- | --- | --- | --- | --- | --- | --- |
|  | **Poor** | **Average** | **Good** | **p-value** | **Poor** | | **Average** | **Good** | **p-value** |
| **Age (in years)** | | | |  | | | | |  |
| 40 years or less | 87 (83.65) | 16 (15.38) | 1 (0.96) | 0.43 | 67 (64.42) | | 31 (29.81) | 6 (5.77) | 0.49 |
| 41-50 | 68 (80) | 16 (18.82) | 1 (1.18) |  | 56 (65.88) | | 26 (30.59) | 3 (3.53) |  |
| More than 50 | 10 (76.92) | 2 (15.38) | 1 (7.69) |  | 7 (53.85) | | 4 (30.77) | 2 (15.38) |  |
| **Education** | | | |  | | | | |  |
| Minimum Educational qualifications (completed class 8) | 48 (92.31) | 3 (5.77) | 1 (1.92) | 0.06 | 40 (76.92) | | 11 (21.15) | 1 (1.92) | **<0.01** |
| Secondary or Senior Secondary (class 9 to 12) | 84 (78.5) | 22 (20.56) | 1 (0.93) |  | 73 (68.22) | | 28 (26.17) | 6 (5.61) |  |
| Graduate and above | 33 (76.74) | 9 (20.93) | 1 (2.33) |  | 17 (39.53) | | 22 (51.16) | 4 (9.3) |  |
| **Work experience (in years)** | | | |  | | | | |  |
| 5 or less | 25 (80.65) | 6 (19.35) | 0 | 0.57 | 20 (64.52) | | 9 (29.03) | 2 (6.45) | 0.45 |
| 6 – 10 | 23 (71.88) | 8 (25) | 1 (3.12) |  | 16 (50) | | 12 (37.5) | 4 (12.5) |  |
| 11 – 15 | 33 (89.19) | 4 (10.81) | 0 |  | 26 (70.27) | | 10 (27.03) | 1 (2.7) |  |
| > 15 | 84 (82.35) | 16 (15.69) | 2 (1.96) |  | 68 (66.67) | | 30 (29.41) | 4 (3.92) |  |

**Effectiveness of a Capacity-Building Training for Knowledge and Practices of Tobacco Control and Oral Cancer Prevention among the ASHA workers in Varanasi**

**SELF-ADMINISTERED QUESTIONNAIRE**

**Serial No.:** ____________ **Date:** ____________

Thank you for your time and responses.

**I. Name: ____________________**

**II. Age: ____________________**

**III. Educational Qualification: ____________________**

**IV. Work Experience as an ASHA Worker (in years): ____________________**

**Tobacco (Smoking: e.g., Cigarettes/Bidis/Hookah; Chewing: e.g., Gutkha, Khaini, Zarda, Pan Masala) and Oral Cancer-Related Questions**

*(Mark ✓ on all correct answers)*

**Q1. What are the risk factors for oral cancer?**
a. Alcohol consumption
b. Use of tobacco
c. Lack of physical activity
d. Family history of cancer
e. Poor oral hygiene
f. Sharp teeth and ill-fitting dentures
g. Obesity
h. Unhealthy diet (low intake of fresh fruits and vegetables)
i. Others, please specify: ______________
j. Don't know

**Q2. Why do people use tobacco?**
a. For pleasure
b. To relieve stress
c. Under peer pressure
d. To appear mature
e. Out of curiosity
f. Others, please specify: ______________
g. Don't know

**Q3. Tobacco use can cause the following diseases:**
a. Asthma/Respiratory diseases
b. Cataract
c. Cancer
d. Increased risk of TB
e. Heart attack
f. Stroke
g. High blood pressure (BP)
h. Diabetes
i. Gum diseases
j. Low birth weight in newborns
k. Impotence and reduced fertility
l. Others, please specify: ______________
m. Don't know

**Q4. Which types of cancer are caused by tobacco use?**
a. Mouth
b. Larynx (voice box)
c. Esophagus (food pipe)
d. Lungs
e. Stomach
f. Throat
g. Breast
h. Uterus
i. Cervix
j. Bladder
k. Others, please specify: ______________
l. Don't know

**Q5. What types of tobacco cause cancer?**
a. Smoking tobacco (e.g., Cigarette/Bidi/Hookah)
b. Chewing tobacco (e.g., Gutkha/Khaini/Zarda/Pan Masala)
c. Areca nut (Supari)
d. None
e. Don't know

**Q6. Can passive smoking (inhaling smoke from others' cigarettes) cause any of the following diseases?**
a. Heart disease
b. Lung disease/cancer
c. Premature birth in babies
d. Others, please specify: ______________
e. Don't know
f. No

**Q7. Why do people find it difficult to quit tobacco?**
a. Addiction
b. Lack of awareness about harmful effects
c. No current health problems
d. No counseling by health professionals
e. Others, please specify: ______________
f. Don't know

**Q8. Have you heard about the COTPA 2003 law?**
a. Yes
b. No

**Q9. Can tobacco products be sold to people under 18 years of age?**
a. Yes
b. No
c. Don't know

**Q10. Is smoking completely prohibited in public places (such as hospitals, public transport like buses/trains, schools/colleges, parks, etc.)?**
a. Yes
b. No
c. Don't know

**Q11. Can tobacco be sold within 100 meters of educational institutions (like schools and colleges)?**
a. Yes
b. No
c. Don't know

**Q12. Are advertisements of tobacco products banned in electronic (TV/Radio) and print media (newspapers)?**
a. Yes
b. No
c. Don't know

**Q13. What are the symptoms of oral cancer?**
a. White/red patches in the mouth
b. Non-healing ulcers/wounds
c. White patches on the inner cheeks
d. Difficulty eating spicy food
e. Difficulty opening the mouth
f. Difficulty sticking out the tongue
g. Voice changes
h. Excessive salivation
i. Difficulty chewing/swallowing/speaking
j. Others, please specify: ______________
k. Don't know

**Q14. Have you ever received training for oral cancer screening?**
a. Yes
b. No

**Q15a. Do you conduct oral cancer screenings in your area?**
a. Always
b. Mostly
c. Sometimes
d. Never

**Q15b. If you conduct screenings sometimes or never, what are the reasons?**
a. Lack of time
b. No training received for screening
c. People are unwilling to get screened
d. Others, please specify: ______________
e. I always/mostly conduct screenings

**Q16. Do you counsel tobacco users in your area to quit tobacco?**
a. Always
b. Mostly
c. Sometimes
d. Never

**Q17. Do you know about the Tobacco Quitline helpline?**
a. Yes
b. No

**Thank you for your time and responses.**
